# The Impact of Isotonic Seawater on Subjective and Objective Nose Patency in Athletes: A Randomized Controlled Trial

**DOI:** 10.3390/jcm14082742

**Published:** 2025-04-16

**Authors:** Andro Košec, Tomislav Vlahović, Branko Šilović, Mislav Rakić, Ana Starešinić, Vedrana Aljinović-Vučić

**Affiliations:** 1 Department of Otorhinolaryngology and Head and Neck Surgery, University Hospital Center Sestre Milosrdnice, 10000 Zagreb, Croatia; 2 School of Medicine, University of Zagreb, 10000 Zagreb, Croatia; 3 Croatian Football Federation Health Service, 10000 Zagreb, Croatia; vlahovic@hns.family (T.V.); silovic.branko@gmail.com (B.Š.); mislav78@gmail.com (M.R.); staresinic.ana@gmail.com (A.S.); 4 Department of Traumatology, University Hospital Center Sestre Milosrdnice, 10000 Zagreb, Croatia; 5 Department of Physiotherapy, University of Applied Health Sciences, 10000 Zagreb, Croatia; 6 Department of Neurosurgery, University Hospital Center Split, 21000 Split, Croatia; 7 Department of Abdominal Surgery, University Hospital Dubrava, 10000 Zagreb, Croatia; 8 Health Center Ozalj, 47280 Ozalj, Croatia; 9 Medical Affairs Department, Jadran Galenski Laboratorij d.d., 51000 Rijeka, Croatia; vedrana.aljinovic@gmail.com; 10 Department of Basic and Clinical Pharmacology and Toxicology, School of Medicine, University of Rijeka, 51000 Rijeka, Croatia

**Keywords:** nasal patency, isotonic nasal spray, nasal irrigation, athletes, exercise

## Abstract

**Background/Objectives:** Nasal irrigation with isotonic seawater is a known and oft-used treatment for nasal obstruction in patients with acute and chronic nasal inflammatory disease undergoing therapy with intranasal corticosteroids and antihistamine drugs. Nasal patency in healthy athletes is extremely important; however, to date, the effect of isotonic solutions for nasal irrigation in healthy athletes has not been tested. This randomized controlled trial aimed to investigate the potential synergy of physical exercise and nasal isotonic seawater on airflow and the subjective assessment of nasal patency in healthy, high-level athletes. **Methods:** The intervention group included 33 healthy athletes who used an isotonic seawater nasal spray daily, with a control group including 31 healthy athletes who did not use any sprays; both groups underwent identical seven-day training periods. The primary outcome measures were subjective NOSE questionnaire scores and secondary peak nasal inspiratory flow (PNIF) measures, while anthropometric and demographic variables were covariates. **Results:** A significant decrease in subjective nasal resistance scores was observed in the intervention group compared to the control group (binary logistic regression model, *p* = 0.006, RR 7.695), both in the first and second measurement interval. This effect increased with time (Friedman’s two-way analysis of variance, *p* < 0.001). Peak nasal inspiratory flow is positively affected by exercise but not by isotonic seawater spray intervention. **Conclusions:** The effects of nasal isotonic seawater irrigation during intense athletic training are beneficial on subjective nasal patency in the short term, while the effects on objective nasal patency are less clear.

## 1. Introduction

Nasal irrigation is a simple method for removing mucus, crusts, and external debris (pollutants, pathogens, and allergens) from the nasal cavity [1]. The technique is often used in conditions like allergic and nonallergic rhinitis and acute and chronic rhinosinusitis, as well as for nonspecific nasal symptoms and preventing upper respiratory tract infections; nasal irrigation is used alone or in combination with other treatments [2]. It is also included in many guidelines for treating allergic rhinitis, as well as the surgical and non-surgical treatment of rhinosinusitis, showing a significant reduction in nasal decongestant use and disease symptom duration [3,4]. It is commonly used in children, since pediatricians refrain from using both oral and topical decongestants, and its effects have been extensively studied [5,6]. Nasal saline irrigation is also used for 4–8 weeks after endoscopic sinus surgery in order to improve healing and prevent infection until the mucosal lining of the nasal cavity and the sinuses has recovered [7,8]. There are different saline solutions that can be used for nasal irrigation: isotonic or hypertonic, buffered or non-buffered, and with or without dispensers [9].

Athletes are a population at significant, special risk of nasal obstruction, with surveys reporting that up to 56% of Olympic athletes complain of allergic rhinitis hindering performance, a higher percentage than that found in the general population [10]. Athletic performance may be hindered by nasal obstruction, impacting ventilation and sleep, while exacerbating asthma. Airflow dynamics may influence airway function in athletes participating in outdoor sports during heavy pollen seasons and those exposed to cold air [11]. The available evidence suggests that nasal congestion can affect pulmonary response, leading to a cytokine-mediated bidirectional increase in bronchial hyperresponsiveness that may significantly impact athletic performance [12,13].

This study aimed to investigate the effect of nasal isotonic seawater irrigation, dispensed through a nasal spray, on the subjective assessment of nasal patency as a primary outcome—and objective nasal airflow as a secondary outcome—in healthy, high-level athletes.

## 2. Patients and Methods

This is a randomized controlled trial involving 64 healthy high-level athletes using isotonic seawater applied via nasal spray during an intensive seven-day training interval.

### 2.1. Outcome Measures

The primary outcome measures were subjective NOSE (Nasal Obstruction Symptom Evaluation) questionnaire scores; this is an internationally validated scale for assessing nasal obstruction intensity, consisting of 5 claims related to nasal obstruction, each divided into 4-point Likert scales (normal values 2.75–7, with scores >7 indicating clinically relevant nasal obstruction). The secondary outcome measures were objective peak nasal inspiratory flow measurements using a calibrated instrument (PNIF, GM Instruments, with normal values ranging from 130 and 140 L/min for healthy young adults), while anthropometric and demographic variables were covariates [14]. PNIF is a noninvasive, easy-to-perform method that is commonly used to assess nasal patency. It is a physiologic measure indicating the peak nasal airflow in liters per minute achieved during maximal forced nasal inspiration [15]. To reduce test–retest variability and sampling bias due to the influence of lateral nasal wall elasticity (the valve effect), three PNIF measurements were performed at every planned time interval, with the best measurement recorded for data analysis. The measurement was performed by two staff physicians who underwent prior joint training by an otorhinolaryngologist. They did this by applying the device to occlude both nostrils and the mouth, after which subjects attempted to inhale exclusively through the nose with maximal effort.

### 2.2. Study Protocol

The protocol was approved by the appropriate bioethical board, adhering to the Ethical Principles for Medical Research Involving “Human Subjects” adopted by the 18th World Medical Assembly, Helsinki, Finland, in June 1964. These principles were most recently amended by the 64th World Medical Assembly, Fortaleza, Brazil, in October 2013 (25 I-29-I I 13-23-03). This study was pre-registered with ClinicalTrials.gov (Identifier: NCT05948800, 9 July 2023) and designed to comply with CONSORT guidelines [16]. The substance tested was nasal spray Aqua Maris Classic, which is classified as a class IIa medical device according to the EU MDR.

The study recruited participants from 1 March 2023 to 31 March 2024. A study sample of 52 was calculated using G*Power (*t*-test, difference between two independent means), based on an effect size of 0.80, 80% study power, and an alpha error rate of 5%. The minimum significant difference in NOSE score was considered to be 0.8 points, with a reference value of 2.6 ± 1 point, based on published values for healthy individuals. The calculation was based on NOSE scores, since that was the primary endpoint of the study. Taking PNIF into sample size calculations as a primary outcome would require a larger sample. Given the limited number of highest-level football athletes in the national football teams, a larger sample size was not possible.

### 2.3. Inclusion and Exclusion Criteria

This study was performed on athletes of both genders (football players registered with the Croatian Football Federation, CFF) > or =18 years. The eligibility of all participants was assessed based on their demographic and anthropometric data, as well as their medical history. All participants were screened by an otorhinolaryngologist and head and neck surgeon prior to inclusion in the study and were under strict professional medical supervision during the study period.

Inclusion criteria were male and female athletes over 18 years of age who participated in the entire intensive seven-day training camp interval. All participants had to sign written informed consent forms.

Exclusion criteria were strictly maintained and included the following: (a) presence of bacterial or viral acute inflammation of the upper respiratory system; (b) chronic obstruction of one or both nostrils with significant septal deformation; (c) rhinoplasty or nasal septum surgery within one month of the start of the study; (d) presence of prior allergic or respiratory disease, such as asthma or allergic rhinitis; and (e) the use of any concomitant medication that might impact nasal or pulmonary function.

During the study period, the use of any medication related to airway management or nasal patency, such as decongestants or mucolytics, or of nasal dilators that might influence the external and internal nasal valve function, was not allowed.

### 2.4. Group Allocation and Randomization

**Figure 1:**
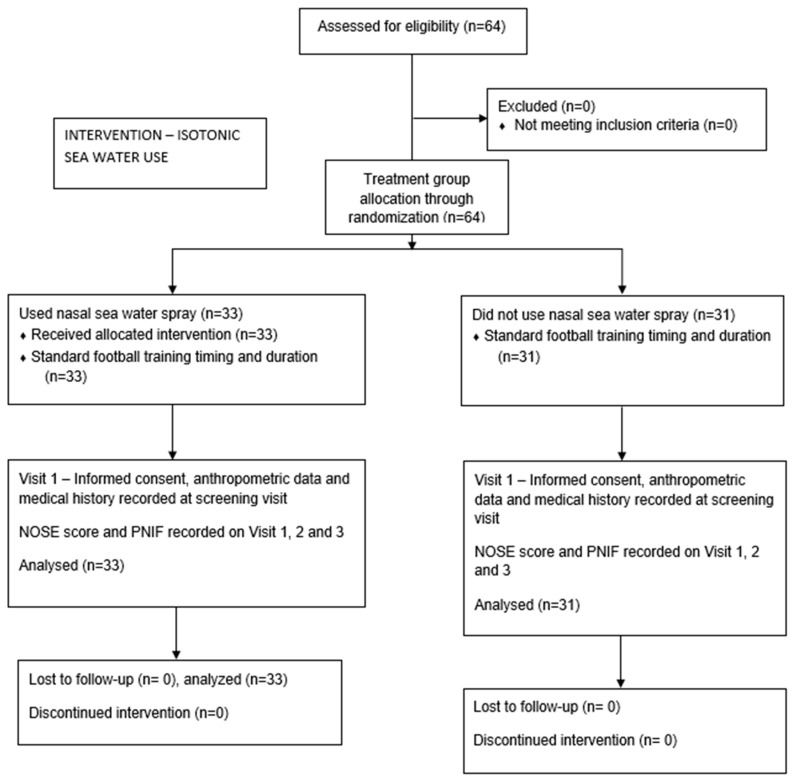
CONSORT study flow diagram.

Participants were randomized into two groups—an intervention and control group, with the substance applied in the former 20 min before exercise three times a day in each nostril throughout a seven-day period. The control group underwent the same exercise plan without using any nasal sprays. Randomization was performed via a binary coin-toss method, with allocation concealment, and using a head–tails method on a true random number generator online service (TRNG). Data collected included age, sex, height, weight, and body mass index as covariates and outcome measures, defined as validated NOSE questionnaire scores (Table 1) and peak inspiratory nasal flow measurement at three-time intervals. These intervals were as follows: (a) Visit 1—basal values before the first training session (first interval), both NOSE and PNIF; (b) Visit 2—after the first spray use and the first training session (second interval), both NOSE and PNIF; and (c) Visit 3—after seven days of thrice daily spray use and after the last training session (third interval), both NOSE and PNIF measurements (Table 2, Figure 1). To ensure participant compliance, a dedicated team of two staff physicians was present during all training sessions, ensuring the intervention group performed adequate nasal spray administration 20 min prior to proceeding with daily practice.

In addition, since nasal airway resistance drops in proportion to exertion, the physiological nasal cycle coupled with exercise may present a confounding effect. However, isocapneic hyperventilation does not alter nasal airflow, indicating that the workload, and not nasal or oral airflow, is the trigger for the nasal and systemic vasoconstrictor response. Body position also does not affect nasal changes in exercise. To reduce test–retest variability and sampling bias due to the influence of lateral nasal wall elasticity, three PNIF measurements were performed at every planned time interval, with the best measurement recorded for data analysis [1].

### 2.5. Statistical Analysis

Statistical analysis was performed, depending on the distribution’s normality, using a binary logistic regression model to compare outcomes between the intervention and control group. Friedman’s two-way test was then performed to assess correlations between measurement intervals (the non-parametric alternative to the one-way ANOVA with repeated measures). A Spearman rho rank correlation test was used to confirm the results obtained from the logistic regression model and Friedman’s test. All statistical significance tests were performed using a two-sided 5% type I error rate.

Statistical analysis was performed using MedCalc software (Version 11.2.1 © 1993–2010. MedCalc Software bvba Software, Broekstraat 52, 9030 Mariakerke, Belgium), and SPSS (Version 22.0., 2013. IBM SPSS Statistics for Windows, Armonk, NY, USA: IBM Corp.) using standard descriptive statistics and frequency tabulation as indicated.

## 3. Results

A total of 64 participants were included in the study, of which 24 were female (giving a male-to-female ratio of 2.66). There were 33 participants in the intervention group using nasal seawater spray irrigation and 31 participants in the control group (Figure 1). The average weight in the cohort was 73.31 kg (SD = 10.18), the average height was 1.78 m (SD = 0.09), and the average BMI value was 22.85 (SD = 1.68).

The average baseline PNIF measures and NOSE score values are presented in Table 3. The measurements at time points 2 (first measurement, after the first training session) and 3 (second measurement, after the last training session) are presented in Table 4 and Table 5, respectively.

Compared to baseline, the total change in PNIF after the second measurement interval was 6.91 L/min (SD = 34.09), while the total change in NOSE score was 0.39 (SD = 1.40) for both groups. For the intervention and control groups, the total PNIF change was 9.1 L/min (SD = 33.1) and 3.7 L/min (SD = 34.5), respectively. Likewise, the total NOSE score change for the intervention group was −0.1 (SD = 0.75), while for the control group it was 0.9 (SD = 1.7).

Compared to baseline scores, the binary logistic regression model showed a significant decrease in subjective nasal patency scores (NOSE score) in the intervention group compared to the control group (*p* = 0.006, RR 7.695), both in the second and third measurement interval.

The Spearman rho rank correlation test showed significant correlations between a lower NOSE score and number of measurements, with the lowest score obtained at the third study interval (−0.285, *p* = 0.023) and every consecutive measurement showing a significantly lower NOSE score compared to the previous interval, regardless of nasal irrigation use (−0.354, *p* = 0.004). A specific significant negative correlation was identified in the congestion section of the NOSE score for the intervention group, showing a link between using nasal irrigation and a lower congestion score in the third study interval (−0.281, *p* = 0.025).

**Figure 2:**
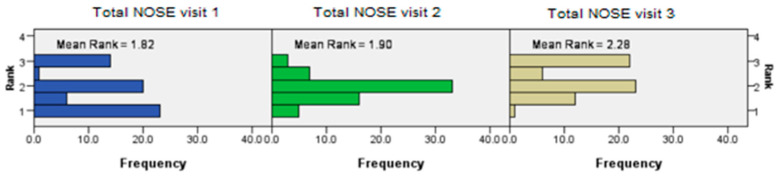
Friedman’s test results showing a significantly larger difference in NOSE score reduction between the test intervals, and that the effect of exercise and isotonic nasal seawater may be synergistic and cumulative.

The significant decrease in subjective nasal patency scores (NOSE score) in the intervention group compared to the control group became more prominent with increasing time intervals, implying that the effect of continuous exercise and isotonic nasal seawater may be synergistic and cumulative (Friedman’s two-way analysis of variance, *p* < 0.002) (Figure 2).

**Figure 3:**
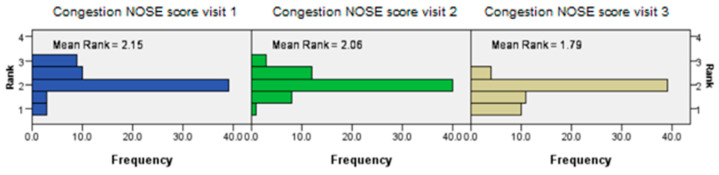
Friedman’s test results showing a significantly lower NOSE score between the test intervals, in favor of the intervention group.

The congestion section of the NOSE score was significantly lower in the intervention group in the third study interval (Friedman’s two-way analysis of variance, *p* < 0.001) (Figure 3).

Peak nasal inspiratory flow was positively affected by exercise in all participants in the second study interval (Friedman’s two-way analysis of variance, *p* < 0.04), but no statistically significant differences were found between the isotonic seawater spray intervention group and the control group. Demographic and anthropometric covariates were not correlated with study outcomes in any of the statistical models.

## 4. Discussion

This randomized controlled trial showed a consistent and significant impact of nasal seawater spray irrigation on subjective nasal obstruction scores in the intervention group versus the control group, while a significant improvement in objective nasal patency measurements was observed in all participants toward the end of the study period.

The results of this study support the routine use of nasal isotonic seawater sprays as a safe and effective measure for improving subjective nasal obstruction, with an effect that seems synergistic with exercise and cumulative over time. The effect of using nasal isotonic seawater irrigation on PNIF changes was not demonstrated, likely owing to the fact that, as previously demonstrated, the nasal airway contributes under 10% to overall ventilation during acute exercise at maximal exercise intensity [6]. However, there are no studies examining the long-term effect of reducing nasal mucosal injury and exercise, a phenomenon that is especially relevant for professional high-level athletes. To reduce test–retest variability and sampling bias due to the influence of lateral nasal wall elasticity, three repeated PNIF measurements were performed at every planned time interval to minimize the impact of internal and external nasal valve function on the results. In addition, we measured the PNIF and NOSE scores at the end of the training sessions, but before the athletes went to shower. This was to prevent additional changes in nasal resistance that might possibly be induced by warm, cold, or hot water and humidity, which could introduce additional bias. In addition, we included male and female athletes performing both indoors and outdoors to control for air humidity and temperature variability. All of these variables impacted the results in the multivariate analysis.

Thus far, few experiments have been performed to evaluate athletes’ training and competition environments and their impact on nasal patency. Whether there are any non-pharmacological interventions useful in treating allergic rhinitis, asthma, and exercise-induced bronchoconstriction in athletes remains unknown [5]. In one study, the impact of an internal nasal dilator on nasal patency was objectively evaluated with anterior rhinomanometry, acoustic rhinometry, and peak nasal inspiratory flow (PNIF). In this study, maximum oxygen uptake (VO2max), maximum pulmonary ventilation, time to exhaustion, and total time of nasal respiration were designated as the primary outcome measures, as assessed by a submaximal treadmill test. Subjective measurements were dyspnea intensity and fatigue perception, evaluated using a labeled visual analog scale. All assessments were performed with and without internal nasal dilator application, which improved aerobic capacity in athletes with compromised nasal valves [17]. However, another study using objective pre- and post-exercise nasal and pulmonary testing showed no significant differences between nasal splinting and normal pre- and post-exercise nasal airflow, suggesting that the nasal valve is not the only factor influencing nasal resistance during exercise [18]. Another study investigated the impact of swimming in indoor pools on nasal patency and pulmonary function, concluding that no significant differences between basal and post-training conditions, as well as no correlation between FEV1 and nasal resistance related to aquatic exercise, were found [19]. One placebo-controlled trial evaluated the relationship between nasal airway patency, aerobic capacity, and exercise workload under nasal occlusion, nasal decongestion, and control test conditions. The authors found no difference in nasal resistance between the decongestant and placebo control groups after exercise, or between resting resistance and after saline nasal spray [20].

Whether changes in nasal resistance have a significant effect on total ventilation and overall respiratory mechanics in athletes has not been definitively shown in the literature. However, the changes that occur in the nasal mucosa, especially when influenced by nasal irrigation during athletic activity, may play an important role in conditioning airflow during exercise and over a wide range of currently unknown and unmeasured parameters in respiratory physiology.

### 4.1. Mechanism of Action of Nasal Irrigation with Seawater

Nasal epithelial dysfunction in athletes may be caused by dehydration and the physical stress applied to the airways during severe exercise hyperpnea, and/or by inhaling noxious agents. This may initiate an inflammatory cascade/repair process that could cause hyperresponsiveness [5]. Nasal breathing at high flow during intense training or competition for a prolonged period of time causes dehydration and upper airway mucosa cooling. This dehydration leads to an increase in inflammatory mediators that ultimately causes airway narrowing [21].

Nasal sprays are more efficient in reducing symptoms than high-volume irrigation. Nasal saline shows a statistically significant reduction in disease symptoms when used as a monotherapy and as an adjunct to other treatment modalities in patients with inflammatory diseases of the nasal cavity [22]. A number of studies have shown that people with different sinonasal conditions can benefit from using both isotonic and hypertonic nasal irrigations when measuring for improved mucociliary clearance, decreased mucosal edema, decreased inflammation mediators, and the mechanical removal of thick mucus and nasal crusts [23,24].

In terms of its composition, seawater is not merely saline (NaCl dissolved in water), since it incorporates four categories of dissolved constituents, which can be defined as major and minor constituents, trace elements, and gases. Seawater has a higher salinity than saline itself, at an average of approximately 35 ppt (parts per thousand). This is due to six major ions: Na, Cl, SO_4_, Mg, Ca, and K. The salinity of the different seawaters differs slightly: between 37 and 38 ppt in smaller seas (such as the Adriatic Sea), lower in open oceans at 33–37 ppt, and as high as 240 ppt in the Dead Sea. However, these different salinities do not affect the relative proportions of major ions, and the average composition is as follows: Cl—55%, Na—30.6%, SO_4_—7.7%, and Mg—4%. The major ions in seawater have been shown to be chemically inactive, and their relative proportion remains practically unchanged over time [4,25].

Nasal irrigation with seawater has both a physical and biological mechanism of action. The physical part is based on the mechanical removal of external material, such as allergens, pathogens, pollutants, and other small particles from the nasal cavity, as well as the removal of secretion material, such as mucus and dried crusts [26]. Furthermore, when hypertonic seawater is applied locally in the nose, it causes water to move through the membrane, resulting in increased water in the nasal cavity. This mechanically cleans the nose and changes mucus consistency from a gel to a liquid, resulting in improved mucociliary transport. At the same time, the quantity of water in the submucosa tissue is decreased, and edema is reduced. The biological mechanism of action is a result of seawater constituents interacting with nasal mucosa cells. Due to these interactions and the favorable effects of ions such as Mg, Ca, K, and HCO_3_, cell viability is increased and inflammation is decreased [27,28].

### 4.2. Clinical Implications and Further Research

Nasal patency is a major training variable for athletes. While there is little significance in the small variations in nasal resistance change during a single exercise session, the effects of nasal dysfunction can have repercussions in the long run, especially in the post-exercise recovery period [6]. Nasal dysfunction is associated with diminished sleep quality and reduced quality of life; it may influence athletic performance in several key areas that are not routinely associated with nasal function. While training protocols are strict, and testing for controlled substances and performance-enhancing drugs is ubiquitous, there are no standard preventive treatments for nasal mucosal injury. This being said, nasal sprays containing isotonic seawater may offer an interesting, safe, cost-effective, and available solution for a significant number of athletes suffering from nasal congestion and/or obstruction. Larger, multicenter studies examining this phenomenon more closely are needed.

### 4.3. Study Limitations

One of the main limitations of this study is that sampling was carried out in three intervals in a single training session. A longer interval was impossible for our single cohort of high-level athletes due to their professional schedules. Although our sample size was adequately powered for the primary outcome measure, we were limited by the total number of available top-level athletes included in the national football teams, and we could not increase the number beyond those available in the male and female top-level teams. In addition, our follow-up was short, thus impacting the long-term implications of our results. The advantage of this study is its robust design, including prospective and randomized measurements accounting for confounding factors and both objective and subjective testing. The novelty of this study lies in its link between intervention and outcomes, showing that this non-pharmaceutical, safe, and tested intervention may positively influence athletic performance by improving nasal patency.

## 5. Conclusions

The effects of nasal isotonic seawater irrigation during intense athletic training are beneficial to subjective nasal patency in the short term. On the other hand, its effects on objective nasal patency are less clear and require a longer period of follow-up with paired objective and subjective outcome measures.

## Figures and Tables

**Table 1:** NOSE questionnaire.

	Not a Problem	Very Mild Problem	Moderate Problem	Fairly Bad Problem	Severe Problem
Nasal congestion or stuffiness					
Nasal blockage or obstruction					
Trouble breathing through my nose					
Trouble sleeping					
Unable to obtain enough air through my nose during exercise or exertion					

**Table 2:** Checklist of the study’s critical time events.

Procedure/Assessment	Visit 1 Day 1 Screening Visit	Visit 2 Day 2	Visit 3 Day 7
Written informed consent	●		
Demographic and anthropometric data	●		
Medical history	●		
Checks for concomitant medications	●		
Check of restrictions	●		
Inclusion/exclusion criteria	●		
Randomization	●		
NOSE	●	●	●
PNIF	●	●	●
Discharge from a clinical study			●

**Table 3:** Overview of mean baseline subjective and objective nasal patency measurements in both study groups at time point 1, before any exercise (SD—standard deviation).

Group/Measurement	Interventional	Control
Baseline PNIF (L/min)	136.5 (SD = 42.6)	
Baseline total NOSE score	5.24 (SD = 4.48)	
Congestion NOSE score	1.19 (SD = 1.15)	
Nasal blockage NOSE score	1.28 (SD = 1.19)	
Troubled breathing NOSE score	1.158 (SD = 1.21)	
Troubled sleeping NOSE score	0.64 (SD = 0.87)	
Inability to breathe during exercise, NOSE score	1 (SD = 1.09)	

**Table 4:** Overview of mean subjective and objective nasal patency measurements in both study groups at time point 2, after the first training session (SD—standard deviation).

Group/Measurement	Interventional	Control	Statistical Significance *p*
PNIF (L/min)	157.1 (SD = 36)	144.2 (SD = 40.7)	
Total NOSE score	3.75 (SD = 3.72)	4.61 (SD = 4.55)	(binary logistic regression, *p* = 0.006)
Congestion NOSE score	1.03 (SD = 0.97)	1.16 (SD = 0.91)	Friedman’s two-way analysis of variance, *p* < 0.001
Nasal blockage NOSE score	0.9 (SD = 0.99)	0.97 (SD = 1.15)	
Troubled breathing NOSE score	0.63 (SD = 0.97)	0.9 (SD = 1.17)	
Troubled sleeping NOSE score	0.36 (SD = 0.64)	0.61 (SD = 0.97)	
Inability to breathe during exercise NOSE score	0.76 (SD = 0.85)	1.03 (SD = 1.09)	

**Table 5:** Overview of mean subjective and objective nasal patency measurements in both study groups at time point 3, after the last training session (SD—standard deviation).

Group/Measurement	Interventional	Control	Statistical Significance *p*
PNIF (L/min)	148.6 (SD = 39.7)	134.7 (SD = 43.4)	Friedman’s two-way analysis of variance, *p* < 0.04
Total NOSE score	2.78 (SD = 3.91)	4.61 (SD = 4.61)	(binary logistic regression, *p* = 0.006)
Congestion NOSE score	0.67 (SD = 1)	0.97 (SD = 1.06)	Friedman’s two-way analysis of variance, *p* < 0.001
Nasal blockage NOSE score	0.73 (SD = 1.05)	0.97 (SD = 1.15)	
Troubled breathing NOSE score	0.60 (SD = 0.98)	0.87 (SD = 1.09)	
Troubled sleeping NOSE score	0.39 (SD = 0.69)	0.61 (SD = 0.9)	
Inability to breathe during exercise, NOSE score	0.42 (SD = 0.74)	1 (SD = 1.16)	

## Data Availability

Data will be made available upon reasonable request from the corresponding author.
